# Supplementary material for: Sri Lankan traditional parboiled rice: A panacea for hyperglycaemia?
Source: PLoS One. 2022 Sep 15;17(9):e0273386. doi: 10.1371/journal.pone.0273386 (PMC9477285; doi:10.1371/journal.pone.0273386)

PLOS ONE

Full title: Sri Lankan traditional parboiled rice: a panacea for hyperglycaemia?

--Manuscript Draft--

**Manuscript Number:**	PONE-D-22-14882
**Article Type:**	Research Article
**Full Title:**	Full title: Sri Lankan traditional parboiled rice: a panacea for hyperglycaemia?
**Short Title:**	Glycaemic response of Sri Lankan traditional parboiled rice
**Corresponding Author:**	Sagarika Ekanayake, Ph D<br>University of Sri Jayewardenepura Faculty of Medical Sciences<br>Kotte, SRI LANKA
**Keywords:**	Sri Lankan traditional rice; parboiling; polishing; nutrient compositional changes; comparative glycaemic indices; resistant starch
**Abstract:**	Abstract<br>The aim of the research was to scientifically prove that parboiled Sri Lankan traditional rice produce lower glycaemic responses comparative to raw unpolished or polished rice. Thus the proximate composition and glycaemic indices (GI) of raw, raw polished and parboiled traditional Sri Lankan rice (Oryza sativa L.) varieties Godaheenati, Batapola el, Dik wee, Dahanala, Unakola samba and Hangimuththan were studied as comparative data are not available. Cooked parboiled rice contained significantly high moisture (P<0.05) than raw or raw polished. Mineral content was low (<1.5%) regardless of processing. Crude protein was comparatively high (5.8-11.0% DM) with 2.1-5% (DM) fat with raw unpolished and parboiled having higher contents. Digestible starch of raw polished was highest compared to parboiled or raw unpolished (68.8-90.5% DM). Resistant starch was significantly (P<0.05) high in parboiled rice (1.1-7.2%) with least total dietary fibre in raw polished rice. All varieties of parboiled and raw polished were found to elicit low GI and high GI respectively. High moisture, high resistant starch and low starch in cooked parboiled rice contributed to low GI compared to raw unpolished or raw polished rice.
**Order of Authors:**	Anuradhi Thennakoon, M Phil
	Sagarika Ekanayake, Ph D
**Additional Information:**	
**Question**	**Response**
**Financial Disclosure**<br><br>Enter a financial disclosure statement that describes the sources of funding for the work included in this submission. Review the submission guidelines for detailed requirements. View published research articles from *PLOS ONE* for specific examples.<br><br>This statement is required for submission and **will appear in the published article** if the submission is accepted. Please make sure it is accurate.	This work was supported by the University of Sri Jayewardenepura, Sri Lanka under grant ASP/01/RE/MED/2016/51 received by SE.<br><br>The funders had no role in study design, data collection and analysis, decision to publish, or preparation of the manuscript.

**Unfunded studies**<br>Enter: *The author(s) received no specific funding for this work.*<br><br>**Funded studies**<br>Enter a statement with the following details:<br>• Initials of the authors who received each award<br>• Grant numbers awarded to each author<br>• The full name of each funder<br>• URL of each funder website<br>• Did the sponsors or funders play any role in the study design, data collection and analysis, decision to publish, or preparation of the manuscript?<br>• **NO** - Include this sentence at the end of your statement: *The funders had no role in study design, data collection and analysis, decision to publish, or preparation of the manuscript.*<br>• **YES** - Specify the role(s) played.<br><br>* typeset	
**Competing Interests**<br><br>Use the instructions below to enter a competing interest statement for this submission. On behalf of all authors, disclose any competing interests that could be perceived to bias this work—acknowledging all financial support and any other relevant financial or non-financial competing interests.<br><br>This statement is required for submission and **will appear in the published article** if the submission is accepted. Please make sure it is accurate and that any funding sources listed in your Funding Information later in the submission form are also declared in your Financial Disclosure statement.<br><br>View published research articles from *PLOS ONE* for specific examples.	The authors have declared that no competing interests exist.

**NO authors have competing interests**<br><br>Enter: *The authors have declared that no competing interests exist.*<br><br>**Authors with competing interests**<br><br>Enter competing interest details beginning with this statement:<br><br>*I have read the journal's policy and the authors of this manuscript have the following competing interests: [insert competing interests here]*<br><br>* typeset	
**Ethics Statement**<br><br>Enter an ethics statement for this submission. This statement is required if the study involved:<br><br>• Human participants<br>• Human specimens or tissue<br>• Vertebrate animals or cephalopods<br>• Vertebrate embryos or tissues<br>• Field research<br><br>Write "N/A" if the submission does not require an ethics statement.<br><br>General guidance is provided below. Consult the submission guidelines for detailed instructions. **Make sure that all information entered here is included in the Methods section of the manuscript.**	Ethical clearance for the study was obtained from the Ethical Review Committee of the Faculty of Medical Sciences, University of Sri Jayewardenepura with the reference number of 72/17. Informed written consent was obtained from each volunteer prior to the study.

Powered by Editorial Manager® and ProduXion Manager® from Aries Systems Corporation

**Format for specific study types**<br><br>**Human Subject Research (involving human participants and/or tissue)**<br>• Give the name of the institutional review board or ethics committee that approved the study<br>• Include the approval number and/or a statement indicating approval of this research<br>• Indicate the form of consent obtained (written/oral) or the reason that consent was not obtained (e.g. the data were analyzed anonymously)<br><br>**Animal Research (involving vertebrate animals, embryos or tissues)**<br>• Provide the name of the Institutional Animal Care and Use Committee (IACUC) or other relevant ethics board that reviewed the study protocol, and indicate whether they approved this research or granted a formal waiver of ethical approval<br>• Include an approval number if one was obtained<br>• If the study involved *non-human primates*, add *additional details* about animal welfare and steps taken to ameliorate suffering<br>• If anesthesia, euthanasia, or any kind of animal sacrifice is part of the study, include briefly which substances and/or methods were applied<br><br>**Field Research**<br><br>Include the following details if this study involves the collection of plant, animal, or other materials from a natural setting:<br>• Field permit number<br>• Name of the institution or relevant body that granted permission	
**Data Availability**<br><br>Authors are required to make all data underlying the findings described fully available, without restriction, and from the time of publication. PLOS allows rare exceptions to address legal and ethical concerns. See the PLOS Data Policy and FAQ for detailed information.	Yes - all data are fully available without restriction

A Data Availability Statement describing where the data can be found is required at submission. Your answers to this question constitute the Data Availability Statement and **will be published in the article**, if accepted.<br><br>**Important:** Stating 'data available on request from the author' is not sufficient. If your data are only available upon request, select 'No' for the first question and explain your exceptional situation in the text box.<br><br>Do the authors confirm that all data underlying the findings described in their manuscript are fully available without restriction?	
**Describe where the data may be found in full sentences. If you are copying our sample text, replace any instances of XXX with the appropriate details.**<br><br>• If the data are **held or will be held in a public repository**, include URLs, accession numbers or DOIs. If this information will only be available after acceptance, indicate this by ticking the box below. For example: *All XXX files are available from the XXX database (accession number(s) XXX, XXX.*).<br>• If the data are all contained **within the manuscript and/or Supporting Information files**, enter the following: *All relevant data are within the manuscript and its Supporting Information files.*<br>• If neither of these applies but you are able to provide **details of access elsewhere**, with or without limitations, please do so. For example:<br><br>*Data cannot be shared publicly because of [XXX]. Data are available from the XXX Institutional Data Access / Ethics Committee (contact via XXX) for researchers who meet the criteria for access to confidential data.*<br><br>*The data underlying the results presented in the study are available from (include the name of the third party*	All relevant data are within the manuscript.

and contact information or URL).<br>• This text is appropriate if the data are owned by a third party and authors do not have permission to share the data.<br><br>* typeset	
Additional data availability information:	

Manuscript

Click here to access/download;Manuscript;Parboiled rice GI 2022 May 23.docx

Full title: Sri Lankan traditional parboiled rice: a panacea for hyperglycaemia?

Short title: Glycaemic response of Sri Lankan traditional parboiled rice

T.P.A.U. Thennakoon, S. Ekanayake*

Department of Biochemistry, Faculty of Medical Sciences, University of Sri Jayewardenepura, Sri Lanka

* Corresponding author

E-mail: sagarikae@sjp.ac.lk; sagarikae@hotmail.com (SE)

Both authors contributed equally to this work

Abstract

The aim of the research was to scientifically prove that parboiled Sri Lankan traditional rice produce lower glycaemic responses comparative to raw unpolished or polished rice. Thus the proximate composition and glycaemic indices (GI) of raw, raw polished and parboiled traditional Sri Lankan rice (*Oryza sativa L.*) varieties *Godaheenati*, *Batapola el*, *Dik wee*, *Dahanala*, *Unakola samba* and *Hangimuththan* were studied as comparative data are not available. Cooked parboiled rice contained significantly high moisture ($P<0.05$) than raw or raw polished. Mineral content was low (<1.5%) regardless of processing. Crude protein was comparatively high (5.8-11.0% DM) with 2.1-5% (DM) fat with raw unpolished and parboiled having higher contents. Digestible starch of raw polished was highest compared to parboiled or raw unpolished (68.8-90.5% DM). Resistant starch was significantly ($P<0.05$) high in parboiled rice (1.1-7.2%) with least total dietary fibre in raw polished rice. All varieties of parboiled and raw polished were found to elicit low GI and high GI respectively. High moisture, high resistant starch and low starch in cooked parboiled rice contributed to low GI compared to raw unpolished or raw polished rice.

1. Introduction

Rice (*Oryza sativa* L.) is the staple food of more than half of the orld's seven billion population [1]. Daily per capita rice consumption is highest in Asia with an intake of more than 300g (>110 kg per capita annually) while India and China account for 50% of the world's rice consumption [2]. Staple rice is also the major source of carbohydrate and protein for Sri Lankans with a per capita consumption of 114 kg/ year [3]. Sri Lankan adults obtain 72% of daily energy requirement from carbohydrate which is mainly from rice [4].

Like world over, Sri Lanka has been witnessing a rapid increase in non-communicable diseases while risk factors such as dysglycaemia, dyslipidaemia and hypertension have shown

a steep incline in past few decades [5]. A positive correlation between intake of refined raw white rice and risk of type 2 diabetes mellitus is proven with higher risk in Asians compared to Western populations [6]. Consumption of refined raw white rice contributes largely to the dietary glycaemic load and higher postprandial glucose levels leading to modifiable risk factors and ultimately non-communicable diseases [7]. Consequently, consumption of whole grains or brown rice is recommended in a healthy diet [8]. Therefore, both the quantity and quality of starch present in frequently consumed carbohydrate rich foods require prudent consideration.

Glycaemic index (GI) is the blood glucose response to an ingested quantity of carbohydrate in a food as compared to the response using a standard reference food [9]. Frequent incorporation of foods with high GI in meals increase the postprandial glucose response and ultimately resulting in insulin resistance [10]. Epidemiological studies have clearly indicated that low GI diets with higher nutritional value decrease the risk of non-communicable diseases, such as type 2 diabetes, coronary heart disease etc [11].

During the past decade a trend in increased consumption of Sri Lankan traditional rice varieties due to the high nutritional and medicinal properties [12,13] is observed. Research have proven that Sri Lankan traditional rice varieties possess high nutritional value when compared to improved varieties [14]. In addition, post-harvest treatment of rice significantly changes the postprandial blood glucose response [15]. Post- harvest processes include milling, parboiling and storing. Rice milling stage starts with de-husking and ends with polishing to remove bran. Whole grain brown rice retains 100% of its bran and germ [16] and is rich in dietary fibre, vitamins, minerals and phenolic compounds [17]. Loss of nutrients during the milling process results in an endosperm-rich grain with easily digestible starch [18]. Thus, raw under milled rice compared to raw milled rice have higher nutritional quality [16].

Limited comparative research had been conducted on effect of different processing methods such as parboiling and polishing on proximate composition and glycaemic indices of Sri Lankan traditional rice. Such information is valuable for consumers, rice cultivators and nutritionists to formulate rice-based diets and to improve the import potential of these varieties. Hence, the objectives of this study were to investigate the effect of parboiling and polishing on nutritional value and glycaemic indices utilizing six Sri Lankan traditional rice varieties.

2. Materials and methods

2.1. Materials

Paddy samples of *Godaheenati*, *Batapola el*, *Dik wee*, *Dahanala*, *Unakola samba* and *Hangimuththan* purchased from the rice preserving centre (*Paramparika Govi Urumayan Rekime Wyaparaya*), Homagama, Sri Lanka were used in the following analyses. Paddy harvested during *Yala* season (2018) were collected into polythene bags, sealed, labelled and transported on the same day to the laboratory and stored under temperature-controlled conditions until processed.

2.2. Methods

2.2.1. Sample preparation

Raw paddy was dehulled (Satake THU 35B), a portion was polished (4% polishing level) (Satake TM 05) and the other portion utilized after dehulling. Parboiled rice was obtained by immersing paddy in boiling hot water and heating until the paddy grains split open and sun drying as practiced traditionally. Dried parboiled paddy was dehulled but not polished.

Rice samples (dehulled only, polished (4%), parboiled) were washed and cooked for 30 to 60 minutes by adding a known amount of water (350-500 mL) as required for each differently processed rice variety. Thus cooked fresh rice was used in GI study on different days.

Proximate analyses were carried out with cooked, oven dried (Memmert, Germany, 55 ^{o}C, 3-4 days), milled (IKA ® A11 basic, New Zealand) and sieved (100 mesh sieve) rice flour.

2.2.3. Moisture and ash

Moisture of freshly cooked rice grains and rice flour were determined as loss of weight on drying [19]. The weighed samples (0.5000g) were oven dried at 105°C until constant weight. The samples were ignited in the muffle furnace (Hobersal, Barcelona, Spain) at 550 ^{o}C for 5-6 hours for ash determination [19].

2.2.4. Crude protein

Crude protein contents were analysed utilizing the [19] micro Kjeldhal apparatus (Paranas Wagner still). A conversion factor of 5.75 was used to determine the protein content.

2.2.5. Carbohydrates and total dietary fibre

Digestible starch and resistant starch contents were determined by amyloglucosidase/ α-amylase (Megazyme total starch assay kit, Ireland). Samples were incubated at 100°C with α-amylase followed by amyloglucosidase at 50 °C and starch content assayed as glucose following reaction with glucose oxidase colourimetrically (Spectro UV-VIS Auto/UV-2602, USA). Soluble, insoluble and total dietary fibre (TDF) contents were determined (Megazyme total dietary fibre assay kit, Ireland) by digesting the samples with α- amylase, protease and amyloglucosidase.

2.2.6. Crude fat

Samples were digested with 7.7M HCl and fat extracted with petroleum ether and diethyl ether using mojonnier flasks [20].

2.2.7. *In-vivo* glycaemic response

Apparently healthy (n = 25, age 20-30 years) volunteers with BMI in the range of 18.5-23 kg/m^2 and not under medical treatment were selected for the study. Each volunteer was

served the standard twice and each test food once in random order on separate days. Glucoline (gsk Glaxco-Wellcome Ceylon Ltd. Sri Lanka) dextrose monohydrate was used as the standard. The volunteers were served with rice portions corresponding to 50 g digestible carbohydrate with gravy made with coconut milk (100 mL), water (125 mL), onions, curry leaves, garlic, fenugreek, turmeric powder, and salt to be ingested within 15 min. Water (250 mL) was also provided to be ingested with the meal/standard [21-22] method.

Finger prick (AccuCheck pricking device), capillary blood samples were collected at fasting and 30-120 min after ingestion of test/ standard food. Blood glucose was estimated with the glucose oxidase enzymatic kit (GOD-PAP, Biolabo, France). Incremental area under the blood glucose curve (IAUC) was determined for each individual for rice and glucose. GI was calculated as a ratio between the IAUC of the test food and the standard. GI was expressed as the average GI of ten individuals. Glycaemic Load (GL) was estimated by multiplying the GI by the amount of net carbohydrates in each portion [23].

2.2.8. Statistical analyses

Data were presented as mean ±SD proximate composition and glycaemic index data as mean ±SEM. Data were analysed by SPSS 25.0 statistical software (IBM SPSS statistics). Descriptive statistics and ANOVA Tukey's posthoc test at 95% confidence interval was used to find the significances.

2.2.9. Ethical clearance

Ethical clearance for the study was obtained from the Ethical Review Committee of the Faculty of Medical Sciences, University of Sri Jayewardenepura with the reference number of 72/17. Informed written consent was obtained from each volunteer prior to the study.

3. Results and discussion

3.1.Proximate composition of differently processed rice flour

As comparative data on nutrient content in differently processed traditional Sri Lankan cooked rice is scarce we studied the fate of nutrients when processed differently. The nutrient compositions of the differently processed 6 traditional rice varieties are stated in tables 1 and 2. Moisture of cooked rice and rice flour were 61-85% and 4.9- 9.8%. Moisture content of cooked rice varied in the order of parboiled > raw > raw polished (Figure 1). When an edible portion of rice is considered significantly lower ($P \leq 0.05$) moisture was present in raw polished rice followed by raw and parboiled rice of each variety. Thus, comparatively the volume of parboiled rice one could consume will contain highest amount of moisture compared to raw or raw polished rice of each variety. Thus, the available nutrient content of an edible portion of differently processed rice will vary depending on the moisture content of cooked rice. Higher moisture content in cooked parboiled rice could be due to the absorption of water by the starch polymorphs formed during parboiling such as annealed starch, lipid–amylose complexes I and II and retrograded amylopectin and amylose [24]. This is the first report on moisture contents of cooked traditional rice of Sri Lanka which are differently processed.

Figure 1: Moisture contents of differently processed freshly cooked rice (mean±standard deviation) and rice portions containing 50 g of carbohydrate of differently processed cooked rice (White variety: *Unakola samba* and red variety: *Dik wee*)

Table 1: Moisture, ash contents, protein and crude fat contents (mean± SD) of differently processed cooked rice flour g/100g in dry weight (fresh weight basis within parenthesis)

Variety	Moisture content~~± SD~~			Ash content± SD			Protein content± SD			Fat content± SD		
	Raw	Raw polished	Parboiled	Raw	Raw polished	Parboiled	*Raw	*Raw polished	*Parboiled	Raw	Raw polished	Parboiled
Goda heenati	9.8±0.3^{p}	8.8±0.3^{q}	6.5±0.0^{r}	1.33±0.04^{p}	0.76±0.05^{q}	1.21±0.09^{r}	9.0±0.4^{p}<br>(3.3±0.1)	7.8±0.6^{q}<br>(2.8±0.2)	9.5±0.2^{p}<br>(2.1±0.0)	5.0±0.2^{p}<br>(1.7±0.1)	2.6±0.7^{q}<br>(0.9±0.2)	3.8±0.3^{r}<br>(0.8±0.1)
Batapola el	5.7±0.4^{p}	6.4±0.5^{p}	5.8±0.1^{p}	1.23±0.06^{p}	0.83±0.02^{q}	1.28±0.10^{p}	9.2±0.4^{p}<br>(2.9±0.1)	7.4±0.7^{q}<br>(2.8±0.3)	8.5±0.3^{r}<br>(1.5±0.1)	4.3±0.5^{p}<br>(1.3±0.1)	3.1±0.1^{q}<br>(1.2±0.1)	5.0±0.1^{p}<br>(0.9±0.0)
Dik wee	6.9±0.2^{p}	7.0±0.4^{p}	4.9±0.4^{q}	1.26±0.04^{p}	0.84±0.02^{q}	0.98±0.06^{r}	8.9±0.2^{p}<br>(2.8±0.1)	6.6±0.6^{q}<br>(2.3±0.2)	10.5±0.2^{r}<br>(1.6±0.0)	4.0±0.3^{p}<br>(1.3±0.1)	2.7±0.2^{q}<br>(0.9±0.1)	4.3±0.3^{p}<br>(0.7±0.0)
Dahanala	6.0±0.4^{p}	7.0±0.7^{p}	5.3±0.1^{q}	1.18±0.08^{p}	0.83±0.04^{q}	1.24±0.03^{r}	10.5±0.2^{p}<br>(3.7±0.1)	10.1±0.4^{p}<br>(3.5±0.1)	11.9±0.4^{q}<br>(1.9±0.1)	4.1±0.1^{p}<br>(1.5±0.0)	2.9±0.1^{q}<br>(1.0±0.0)	4.0±0.1^{p}<br>(0.7±0.0)
Unakola samba	9.8±0.2^{p}	9.2±0.3^{p}	6.7±0.1^{q}	0.81±0.06^{p}	0.69±0.09^{q}	1.03±0.04^{r}	8.6±0.2^{p}<br>(2.7±0.1)	8.9±0.2^{p}<br>(3.5±0.1)	9.7±0.7^{p}<br>(2.0±0.1)	3.7±0.6^{p}<br>(1.4±0.2)	2.8±0.2^{q}<br>(1.1±0.1)	4.8±0.1^{r}<br>(1.0±0.0)
Hangimuththan	8.0±0.3^{p}	5.2±0.3^{q}	5.6±0.1^{q}	0.84±0.05^{p}	0.68±0.03^{q}	1.15±0.07^{r}	8.3±0.4^{p}<br>(3.1±0.1)	7.6±0.4^{q}<br>(2.7±0.1)	8.3±0.2^{p}<br>(1.7±0.0)	4.2±0.3^{p}<br>(1.3±0.0)	2.1±0.0^{q}<br>(0.7±0.0)	4.9±0.4^{p}<br>(1.0±0.1)

n= 5; *n= 3; SD: Standard Deviation; p, q and r superscripts along a row in each parameter (Moisture, Ash, Protein and Fat) indicate significances among differently processed rice varieties at 95% confidence interval

Table 2: Digestible starch, resistant starch and total dietary fiber contents of differently processed cooked rice flour g/100g in dry weight (fresh weight basis within parenthesis)

Variety	Digestible starch content ± SD			Resistant starch content± SD			Total dietary fiber content ± SD		
	Raw	Raw polished	Parboiled	Raw	Raw polished	Parboiled	*Raw	*Raw polished	*Parboiled
Godaheenati	78.4±2.1[a]<br>(25.7±0.7)	82.1±2.9[a]<br>(29.6±1.1)	76.4±0.4[a]<br>(16.6±0.1)	3.9±0.4[p]	2.5±0.5[q]	6.3±0.2[r]	10.8±0.8[x]	6.4±0.2[y]	11.4±1.0[x]
Batapola el	75.8±3.5[a]<br>(23.6±1.1)	79.0±2.4[a]<br>(29.6±0.9)	73.5±1.6[a]<br>(13.1±0.3)	4.4±0.3[p]	3.3±0.2[q]	6.5±0.7[r]	11.1±0.1[x]	6.1±0.7[y]	11.7±0.3[x]
Dik wee	76.7±0.9[a]<br>(24.0±0.3)	90.5±5.1[b]<br>(31.0±1.7)	74.7±1.4[a]<br>(11.6±0.2)	2.2±0.3[p]	1.1±0.2[q]	6.7±0.2[r]	11.6±0.2[x]	5.8±0.4[y]	11.4±1.0[x]
Dahanala	68.8±3.4[a]	80.7±2.1[b]	73.0±2.5[a]	5.3±0.5[p]	2.1±0.4[q]	7.2±0.5[r]	11.6±0.3[x]	7.2±0.5[y]	10.6±0.4[x]

	(23.4±1.1)	(28.3±0.7)	(11.9±0.4)						
Unakola samba	78.7±0.9^{a}	82.5±1.3^{b}	73.6±2.6^{c}	5.8±0.6^{p}	3.2±0.7^{q}	6.7±0.7^{r}	9.5±0.1^{x}	6.0±0.1^{y}	10.5±1.8^{x}
	(24.7±0.3)	(32.4±0.5)	(15.0±0.5)						
Hangimuththan	79.2±1.4^{a}	82.2±5.8^{a}	76.4±2.1^{a}	4.6±0.3^{p}	2.7±0.4^{q}	6.1±0.4^{r}	9.1±0.5^{x}	5.7±0.8^{y}	10.6±0.4^{x}
	(24.6±0.4)	(29.6±2.1)	(15.2±0.4)						

n= 5; *n= 3; SD: Standard Deviation; a, b and c superscripts along a row indicate significances in digestible starch contents, p, q and r superscripts along a row indicate significances in RS contents and x and y superscripts along a row indicate significances in total dietary fiber contents among differently processed cooked varieties at 95% confidence interval

Ash contents of differently processed cooked rice flour ranged from 0.68- 1.33% respectively with raw polished varieties having significantly lower ($P \leq 0.05$) amounts due to removal of bran compared to raw unpolished and parboiled varieties. The ash contents of milled rice were comparatively lower compared to under-milled rice [25-26]. Similar results have been observed for other under-milled raw traditional Sri Lankan rice varieties [12, 27]. Irrespective of processing two white coloured varieties, *Unakola samba* and *Hangimuththan* had lower ($P \geq 0.05$) ash contents compared to red varieties. Red pericarp rice varieties contain higher content of minerals [28].

Crude protein contents of all differently processed cooked rice flour varied in the range of 7-12% (DW) (Table 1) and similar results (7-13% DW) were observed for under-milled traditional raw rice varieties [12, 27-28]. Except for *Batapola el*, parboiled rice varieties contained higher protein content (DM) compared to raw and raw polished varieties. Traditional rice varieties contained more protein compared to newly improved varieties [29-31]. However, parboiled rice of all varieties had the least protein content on fresh weight (FW) basis due to the comparatively higher moisture (Figure 1). When considering freshly cooked rice the percentage reduction of protein due to parboiling compared to raw rice ranged between 26-49% (FW) with *Dahanala* showing the highest reduction due mainly to high moisture in cooked rice. However, it was noteworthy that parboiled rice flour contained higher protein than raw or raw polished rice flour where parboiling process has contributed in retaining more protein. Thus incorporation of flour of parboiled rice for food preparation will be more nutritional and healthier.

Crude fat contents of differently processed cooked rice ranged from 2.1- 5.0% (DM) (Table 2). Flour of raw polished varieties had significantly lower ($P \leq 0.05$) fat contents compared to raw and parboiled varieties due to the removal of bran layer and germ which are rich in fats whereas under-milled rice and parboiled retains bran and germ which accommodates fat

present in the grain [32]. However, fat content of reported traditional raw rice was less (not exceeding 3.5% DW) when compared to the present study [12,14,27-28]. This could be due to the method of analysis as lipid content of a given sample vary with the method of analysis [33]. Furthermore, the fat content of all differently processed rice on fresh weight basis was less than 1.7% with parboiled rice having the least fat content which is due to the high moisture content.

Digestible carbohydrate comprised more than two thirds of the weight of rice contributing to metabolizable energy. The digestible starch of raw, raw polished and parboiled rice flour ranged between 68-79%, 79-90.5% and 73-76% (DW) respectively (Table 2). Majority of raw polished varieties had significantly higher ($P \leq 0.05$) digestible starch content due to the removal of outer layers which increased the starch contents of rice grain as only the kernel remains. The digestible starch content of parboiled rice per equivalent amount of raw and raw polished rice on fresh weight was significantly ($P \leq 0.05$) less due to higher moisture content. Therefore, comparatively the amount of parboiled rice that one could consume would be less compared to raw or raw polished and thus will contribute to a lower carbohydrate load making these more suitable in the diets of obese and individuals with NCDs.

Resistant starch (RS) contents of differently processed cooked rice flour varied from 1.1-7.2% (Table 2). Cooking increased the RS content in parboiled rice by 6-7 fold, in raw under-milled by 2-5.5 fold and in raw polished by 2-3 fold compared to uncooked rice. A significant difference in differently processed varieties was observed where raw polished varieties had the least ($P \leq 0.05$) and parboiled ($P \leq 0.05$) had the highest resistant starch contents. Resistant starch contents of parboiled varieties of present study were significantly higher confirming the contribution made by retrograded starch (Type 3) to resistant starch due to parboiling process [34]. This further makes traditional parboiled rice more suitable in

the diets of people with NCDs. In addition, the high resistant starch would make parboiled rice an excellent prebiotic food [35].

Total dietary fiber contents of cooked raw, raw polished and parboiled rice flour varied in the range of 9.1-11.6%, 5.7-7.2% and 10.5-11.7% respectively (Table 2) where raw polished varieties had significantly lower (P≤ 0.05) total dietary fiber. Similar data for raw under-milled traditional rice varieties are reported (4.2- 6.9% DW) [12, 27]. The contribution from insoluble dietary fiber in raw, raw polished and parboiled varieties was 8.1-9.3%, 5.3-6.2% and 7.3-9.5% respectively with lower soluble dietary fiber in all differently processed cooked varieties (0.2- 3.2%). Similar to total dietary fiber, significantly low (P≤ 0.05) insoluble and soluble fibre contents were found in raw polished varieties compared to raw or parboiled varieties. As nearly 90% of dietary fiber consists of insoluble dietary fibre (IDF) [36] removal of outer layers could be the reason for the least IDF content in raw polished varieties.

Thus, when considering the nutrients in rice flour, the macronutrient contents were highest in raw unpolished rice followed by parboiled and raw polished rice. Thus consumption of traditional rice is more nutritious as raw unpolished or parboiled rice. The nutrients and dietary fiber reduced significantly (P< 0.05) irrespective of lower level of polishing (4%) rice was subjected to in the present study. Thus subjecting rice to higher level of polishing as available in the market will contribute to increase digestible carbohydrate which is easily available for ~~digestion~~ and rapidly increase glucose and insulin responses.

3.2. Glycaemic responses of differently processed rice

The data related to glycaemic response, glycaemic indices and glycaemic loads of differently processed rice varieties are stated in tables 3 and 4. We describe the effect of processing on traditional Sri Lankan rice in relation to the glycaemic response and the effect the inherent

nutrients have on the said response. This is the first report on such an attempt with Sri Lankan traditional rice to the best of our knowledge.

Portion sizes given for the GI determination varied in a wide range as 140g to 412g (Table 3) where raw polished varieties had the lowest and parboiled varieties had the highest weight (g) (Figure 1). The higher moisture content in cooked parboiled rice (Figure 1) made the portion sizes larger (volume) and difficult to consume.

Table 3: Portion size for GI, peak blood glucose and % peak reduction compared to glucose

Variety	Portion size for GI (g)			Peak glucose concentration (mg/dL)			% Peak reduction		
	Raw	Raw polished	Parboiled	Raw	Raw polished	Parboiled	Raw	Raw polished	Parboiled
Godaheenati	185	154	282	115	117	95	6.1	4.3	28.4
Batapola el	212	158	360	116	120	92	7.8	4.2	35.9
Dik wee	208	150	412	117	120	94	6.8	4.2	33.0
Dahanala	224	164	391	117	120	96	6.0	3.3	29.2
Unakola samba	202	140	333	115	116	93	8.7	7.8	34.4
Hangimuththan	203	160	311	121	124	112	5.8	3.2	14.3

n=10

When the glycaemic curves (Figure 2) were studied the average peak glucose responses were observed at 30-40 minutes following ingestion for all differently processed traditional rice varieties. The incremental area under the curve (IAUC) of all rice varieties were significantly ($P \leq 0.05$) lower than glucose and parboiled rice (1720-2281) had the least area followed by

raw varieties (2553- 3213) with highest in raw polished varieties (3105- 3410). Some parboiled improved and non-parboiled traditional rice produced significantly ($P \leq 0.05$) lower IAUC for all rice varieties when compared to standard glucose [37].

Figure 2: Average glucose response of differently processed a) *Dik wee* (red rice variety) over standard glucose b) *Unakola samba* (white rice variety) over standard glucose (RP-raw polished; PB- parboiled)

Table 4: Glycaemic indices and glycaemic loads of differently processed rice

Processing	Raw		Raw polished		Parboiled	
	GI ± SEM	GL	GI ± SEM	GL	GI ± SEM	GL
Variety						
Godaheenati	58±4	29	76±5	38	45±3	23
Batapola el	54±6	27	76±5	38	47±5	23
Dik wee	69±7	34	70±4	35	43±3	22
Dahanala	60±5	30	69±3	35	48±4	24
Unakola samba	65±8	33	73±5	36	44±3	22
Hangimuththan	55±7	27	74±4	37	43±3	22

n=10

The average peak glucose concentrations were also highest in raw polished (116-124 mg/dL) followed by raw rice (115-121 mg/dL) and parboiled rice (92-112 mg/dL). In contrast, percentage peak reduction was highest in parboiled rice (14.3-34.4%) and least in raw polished rice varieties (5.8-8.7%) in comparison to glucoose. According to Nisanka & Ekanayake [31] parboiled Nadu variety showed lower mean peak glucose concentration followed by raw samba and basmati variety with highest mean peak glucose concentration. Both raw and parboiled rice varieties contained high resistant starch and total dietary fiber

contents (Table 2) compared to raw polished varieties which have contributed to decrease glycaemic indices of raw and parboiled rice.

In accordance with above observations the GI values of raw unpolished, raw polished and parboiled were between 54-69, 69-76 and 43-76 (Table 4). A clear decline in GI (low) of parboiled varieties was observed. Two of the raw unpolished varieties (*Batapolael* and *Hangimuththan*) also elicited low glycaemic index ($\geq$55). Remaining raw varieties (*Godaheenati*, *Dik wee*, *Dahanala* and *Unakola samba*) and one raw polished variety (*Dahanala*) were categorized as medium glycaemic index (56- 69). Except *Dahanala* other raw polished varieties were categorized as high glycaemic index ($\leq$70). The present study data also proved that there is no correlation between glycaemic index and the colour of the pericarp [30, 37]. When considering the effects of inherent nutrients in rice, negative non-significant ($P\leq$ 0.01) correlations between protein, fat, digestible starch and total dietary fiber contents present in 50 g carbohydrate containing portion of raw, raw polished and parboiled rice varieties and their corresponding glycaemic indices were observed. This clearly demonstrated the quantity of inherent nutrients in rice is not adequate to significantly impact the glycaemic index. However, the impact of RS on GI was apparent as a significant negative correlation (P=0.000; r=-0.858) was observed with glycaemic index and RS content of rice portion given to determine the GI of differently processed rice of each variety (Figure 3a).

Figure 3: Correlation between a) Glycaemic index and resistant starch content in the portion ~~give~~ to study the glycaemic index b) Moisture content in the given portion given to study the glycaemic index and portion size

Glycaemic load (GL) values of raw, raw polished and parboiled varieties were in the ranges of 27-34, 35-38 and 22-24 respectively. Thus, the GL for the portions given for glycaemic index determination were categorized as high (>20) for all varieties irrespective of processing. A significant positive correlation (r=0.932; p=0.000) was observed between rice

portion size used to determine the GI and the moisture content in 50g carbohydrate portion (Figure 3b). Therefore, when considering a normal edible portion, GLs of all parboiled varieties which contained high moisture reduced to medium glycaemic load whereas raw polished rice still contained a high glycaemic load.

4. Conclusion

It was apparent from the above results, that processing causes significant changes in nutrient content and glycaemic response of a particular rice variety. Irrespective of variety the moisture content of cooked parboiled was highest which caused to reduce the nutrients but not significantly less when compared to raw rice. In contrast, polished rice contained mainly digestible starch with least amount of protein, fat, dietary fiber and resistant starch. Consequently, parboiled rice elicited lower GI followed by raw and polished rice. The GL of parboiled reduced significantly when an edible portion was considered as the volume of the portion that could be ingested became low due to high moisture retention in parboiled rice. Thus parboiled under milled traditional rice irrespective of variety was nutritionally and health wise more superior for consumption compared to raw or raw polished rice. In addition, flour of parboiled rice with high protein, fat, resistant starch and low carbohydrate would be most suitable for use in food industry as a functional ingredient.

5. Acknowledgment

Volunteers participated in the glycaemic index study are acknowledged.

6. References

1 Muthayya, S., Hall, J., Bagriansky, J., Sugimoto, J., Gundry, D., Matthias, D., Prigge, S., Hindle, P., Moench-Pfanner, R., & Maberly, G. (2012). Rice Fortification: An Emerging Opportunity to Contribute to the Elimination of Vitamin and Mineral Deficiency Worldwide. Food and Nutrition Bulletin, 33(4), 296–307. https://doi.org/10.1177/156482651203300410

2 Muthayya, S., Sugimoto, J. D., Montgomery, S., & Maberly, G. F. (2014). An overview of global rice production, supply, trade, and consumption. Annals of the New York Academy of Sciences, 1324(1), 7–14. https://doi.org/10.1111/nyas.12540

3 Rambukwella, R. & Priyankara, E. A. C. (2016). *Production and marketing of traditional rice varieties in selected districts in Sri Lanka: Present status and future prospects.* Colombo: Hector Kobbekaduwa Agrarian Research and Training Institute.

4 Jayawardena, R., Thennakoon, S., Byrne, N., Soares, M., Katulanda, P., & Hills, A. (2014). Energy and nutrient intakes among Sri Lankan adults. *International Archives of Medicine*, 7(1). https://doi.org/10.1186/1755-7682-7-34

5 Katulanda, P., Ranasinghe, P., Jayawardana, R., Sheriff, R., & Matthews, D. R. (2012). Metabolic syndrome among Sri Lankan adults: Prevalence, patterns and correlates. *Diabetology and Metabolic Syndrome*, 4(1). https://doi.org/10.1186/1758-5996-4-24

6 Hu, E. A., Pan, A., Malik, V., & Sun, Q. (2012). White rice consumption and risk of type 2 diabetes: meta-analysis and systematic review. *British Medical Journal*, *344*, e1454–e1454. https://doi.org/10.1136/bmj.e1454

7 Blaak, E. E., Antoine, J.-M., Benton, D., Björck, I., Bozzetto, L., Brouns, F., Diamant, M., Dye, L., Hulshof, T., Holst, J. J., Lamport, D. J., Laville, M., Lawton, C. L., Meheust, A., Nilson, A., Normand, S., Rivellese, A. A., Theis, S., Torekov, S. S., & Vinoy, S. (2012). Impact of postprandial glycaemia on health and prevention of disease. *Obesity Reviews*, 13(10), 923–984. https://doi.org/10.1111/j.1467-789X.2012.01011.x

8 WHO | Noncommunicable diseases country profiles 2018. (2018). Retrieved from https://www.who.int/news-room/fact-sheets/detail/noncommunicable-diseases. Accessed July 01 2021.

9 Jenkins, D. J., Wolever, T. M., Taylor, R. H., Barker, H., Fielden, H., Baldwin, J. M., Bowling, A. C., Newman, H. C., Jenkins, A. L., & Goff, D. V. (1981). Glycemic index of foods: a physiological basis for carbohydrate exchange. *The American Journal of Clinical Nutrition*, 34(3), 362–366. https://doi.org/10.1093/ajcn/34.3.362

10 Augustin, L. S., Franceschi, S., Jenkins, D. J., Kendall, C. W., & La Vecchia, C. (2002).

Glycemic index in chronic disease: a review. *European journal of clinical nutrition,* 56(11), 1049–1071. https://doi.org/10.1038/sj.ejcn.1601454

11 Clar, C., Al-Khudairy, L., Loveman, E., Kelly, S. A., Hartley, L., Flowers, N., Germanò, R., Frost, G., & Rees, K. (2017). Low glycaemic index diets for the prevention of cardiovascular disease. *The Cochrane database of systematic reviews,* 7(7), CD004467. https://doi.org/10.1002/14651858.CD004467.pub3

12 Abeysekera, W. K. S. M., Arachchige, S. P. G., Ratnasooriya, W. D., Chandrasekharan, N. V., & Bentota, A. P. (2017). Physicochemical and nutritional properties of twenty three traditional rice (Oryza sativa L.) varieties of Sri Lanka. *Journal of Coastal Life Medicine, August*, 343–349. https://doi.org/10.12980/jclm.5.2017j7-59.

13 Withanawasam, D.M. (2017). *Heritage of Heirloom Rice varieties of Sri Lanka,* Retrieved from https://www.doa.gov.lk/rrdi/images/Mydoc/ from publication1.pdf. Acceessed July 01, 2021

14 Kariyawasam, T. I., Godakumbura, P. I., Prashantha, M. A. B., & Premakumara, G. A. S. (2016). Proximate Composition, Calorie Content and Heavy Metals (As, Cd, Pb) of Selected Sri Lankan Traditional Rice (Oryza Sativa L.) Varieties. *Procedia Food Science*, 6, 253–256. https://doi.org/10.1016/j.profoo.2016.02.036

15 Foster-Powell, K., Holt, S. H., & Brand-Miller, J. C. (2002). International table of glycemic index and glycemic load values: 2002. *The American Journal of Clinical Nutrition*, 76(1), 5–56. https://doi.org/10.1093/ajcn/76.1.5

16 Juliano, B. O. (1993). *AO Food and Nutrition Series No. 26 RICE in human nutrition Prepared in collaboration with FAO by*. http://books.irri.org/9251031495_content.pdf

17 Mosaed, F., Al-Subhi, M., & Hejazi, M. A. (2013). Brown Rice as a Healthy Food and Lowering Blood Sugar in Rats. *Journal of American Science, 9*(4),21-28.

18 Mohan, V., Ruchi, V., Gayathri, R., Ramya Bai, M., Shobana, S., Anjana, R. M., Unnikrishnan, R., & Sudha, V. (2017). Hurdles in Brown Rice Consumption. *Brown Rice,* 255–269. *Springer International Publishing*. https://doi.org/10.1007/978-3-319-59011-0_15

19 Association of Official Analytical Chemists (2000). *Official Methods of Analysis of the*

AOAC. 17th ed.; AOAC International. Maryland, USA.

20 Croon, L.B and Fuchs, G (1980). Crude fat analysis of different flours and flour products. *Var Foda*. 32, 425-427.

21 FAO/WHO. (1998). *Carbohydrates in Human Nutrition: Report of a Joint FAO/WHO Expert Consultation*, 14-18 April 1997, Rome. FAO Food and Nutrition Paper No. 66. Rome.

22 Brouns, F., Bjorck, I., Frayn, K. N., Gibbs, A. L., Lang, V., Slama, G., & Wolever, T. M. S. (2005). Glycaemic index methodology. *Nutrition Research Reviews*, 18(01), 145. https://doi.org/10.1079/NRR2005100

23 Salmeron, J., Manson, J. E., Stampfer, M. J., Colditz, G. A., Wing, A. L., Willett, W. C. (1997). Dietary fiber, glycemic load, and risk of non-insulin-dependent diabetes mellitus in women. *Journal of the American Medical Association*, 277, 472 –477. doi: 10.1001/jama.1997.03540300040031.

24 Bhattacharya, K. R. (2013). Effect of parboiling on rice quality. *Rice Quality*, 247–297. Elsevier. https://doi.org/10.1533/9780857092793.247

25 Sompong, R., Siebenhandl-Ehn, S., Linsberger-Martin, G., & Berghofer, E. (2011). Physicochemical and antioxidative properties of red and black rice varieties from Thailand, China and Sri Lanka. *Food Chemistry*, *124*(1), 132–140. https://doi.org/10.1016/j.foodchem.2010.05.115

26 Trinidad, T. P., Mallillin, A. C., Encabo, R. R., Sagum, R. S., Felix, A. D., & Juliano, B. O. (2013). The effect of apparent amylose content and dietary fiber on the glycemic response of different varieties of cooked milled and brown rice, *International Journal of Food Sciences and Nutrition*, *64*(1), 89–93. https://doi.org/10.3109/09637486.2012.700922

27 Samaranayake, M. D. W., Yathursan, S., Abeysekera, W. K. S. M., & Herath, H. M. T. (2017). Nutritional and antioxidant properties of selected traditional rice (Oryza sativa L.) varieties of Sri Lanka. *Sri Lankan Journal of Biology*, *2*(2), 25. https://doi.org/10.4038/sljb.v2i2.10

28 Kulasinghe, A., Samarasinghe, G., Wimalasiri, S., Silva, R., & Madhujith, T. (2019). Macronutrient and mineral composition of selected traditional rice varieties in Sri Lanka.

Proceedings of the International Conference on Food Quality, Safety and Security 1, 1–8.

29 Gunaratne, A., Bentota, A., Cai, Y. Z., Collado, L., & Corke, H. (2011). Functional, digestibility, and antioxidant properties of brown and polished rice flour from traditional and new-improved varieties grown in Sri Lanka. *Starch/Staerke*, 63(8), 485–492. https://doi.org/10.1002/star.201000105

30 Hettiarachchi, P., Jiffry, M., Jansz, E., Wickramasinghe, A., & Fernando, D. (2014). Glycaemic indices of different varieties of rice grown in Sri Lanka. *Ceylon Medical Journal*, 46(1), 11. https://doi.org/10.4038/cmj.v46i1.6516

31 Nisanka, T. U. S., & Ekanayake, S. (2016). Rice variety and processing: contribution to glycaemic response. *Ceylon Medical Journal*, 61(4), 159. https://doi.org/10.4038/cmj.v61i4.8381

32 Chapagai, M. K., Wan Rosli, W. I., Wan Manan, W. M., Jalil, R. A., Karrila, T., & Pinkaew, S. (2017). Effect of domestic cooking methods on physicochemical, nutritional and sensory properties of different varieties of brown rice from Southern Thailand and Malaysia. *International Food Research Journal*, 24(3), 1140–1147.

33 Zhou, Z., Robards, K., Helliwell, S., & Blanchard, C. (2002). Composition and functional properties of rice. *International Journal of Food Science and Technology*, *37*(8), 849–868. https://doi.org/10.1046/j.1365-2621.2002.00625.x

34 Gunaratne, A., Kao, W., Ratnayaka, J., Collado, L.& Corke, H. (2013). Effect of parboiling on the formation of resistant starch, digestibility and functional properties of rice flour from different varieties grown in Sri Lanka. *Journal of the Science of Food and Agriculture* 93(11), 2723–2729. https://doi.org/10.1002/jsfa.6091

35 Topping, D., Fukushima, M., & Bird, A. (2003). Resistant starch as a prebiotic and synbiotic: state of the art. *The Proceedings of the Nutrition Society*. 62. 171-176. https://doi.org/10.1079/PNS2002224.

36 Zhao, G., Zhang, R., Dong, L., Huang, F., Tang, X., Wei, Z., & Zhang, M. (2018). Particle size of insoluble dietary fibre from rice bran affects its phenolic profile, bioaccessibility and

functional properties. *LWT - Food Science and Technology*, *87*, 450–456. https://doi.org/10.1016/j.lwt.2017.09.016

37 Pathiraje, P., Madhujith, W., Chandrasekara, A., & Nissanka, S. (2010). The Effect of Rice Variety and Parboiling on in vivo Glycemic Response. *Tropical Agricultural Research*, 22(1), 26. https://doi.org/10.4038/tar.v22i1.2667

Figure 1

Click here to access/download;Figure;Figure 1.tif

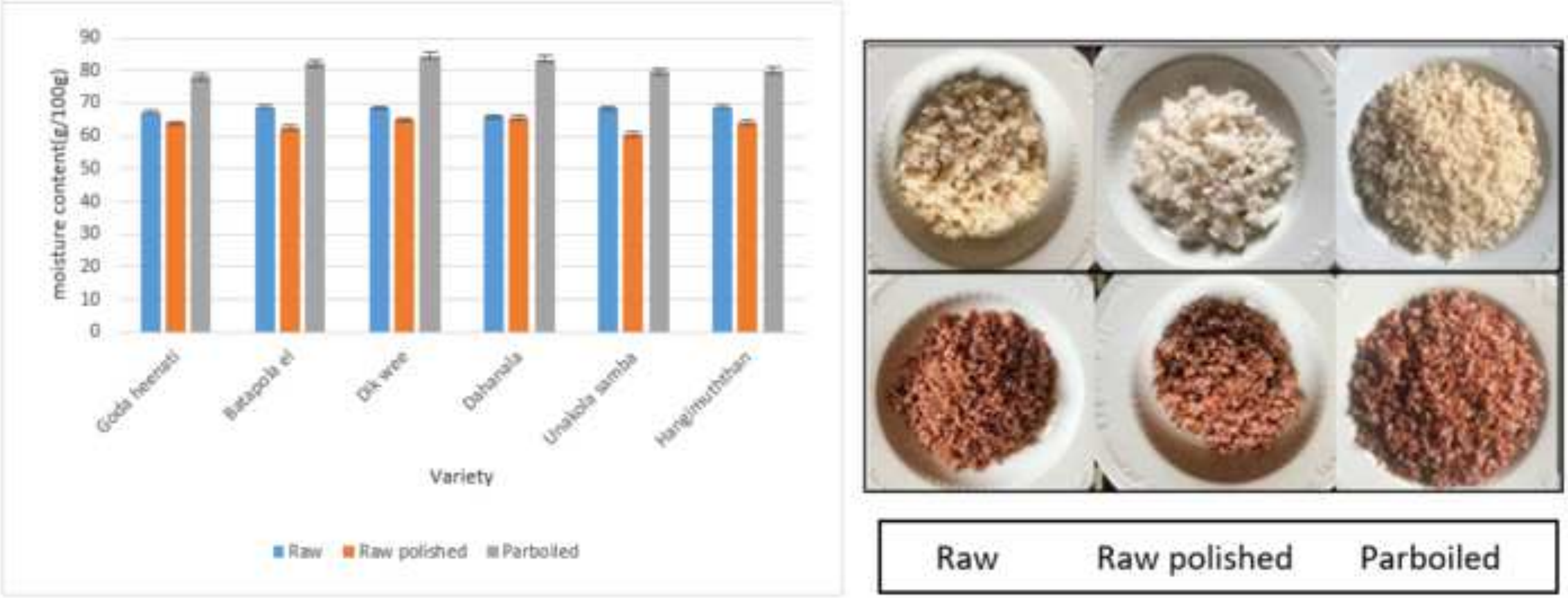

Figure 2

Click here to access/download;Figure;Figure 2.tif

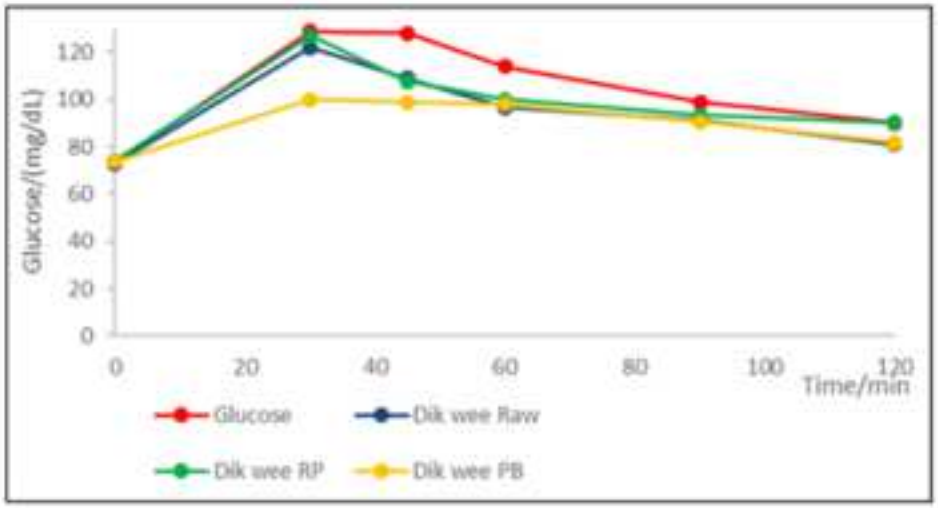

a)

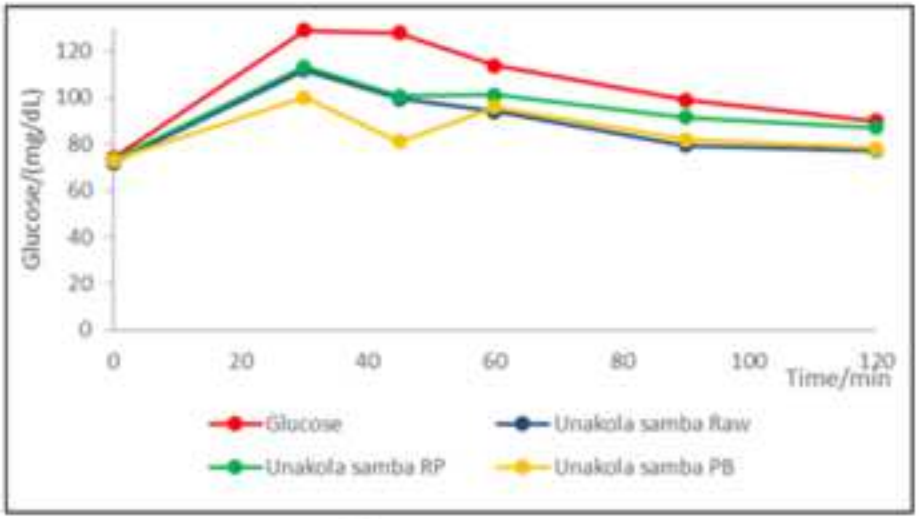

b)

Click here to access/download;Figure;Figure 3.tiff

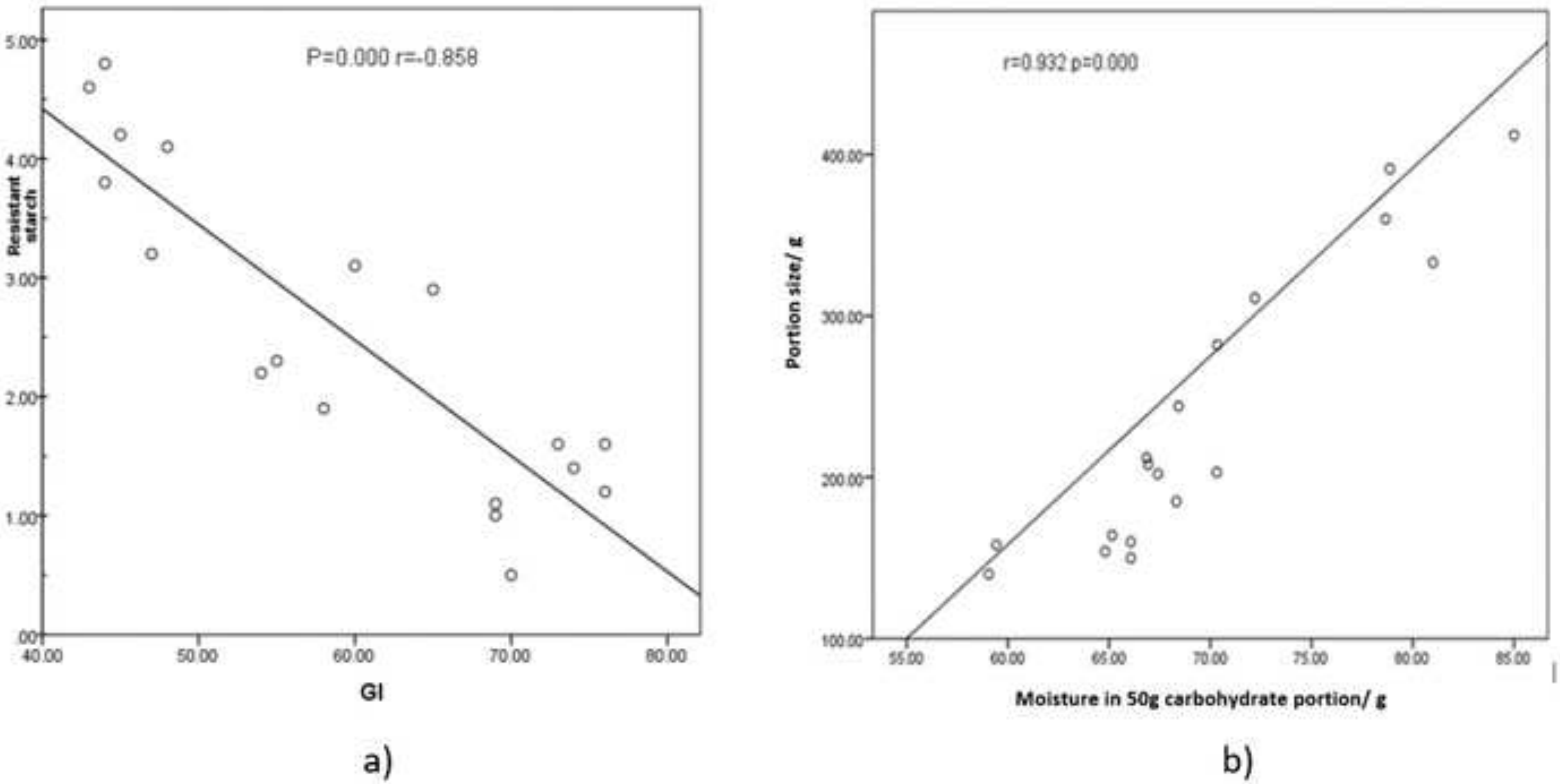

Supplement: Attachment — Submitted filename: PONE-D-22-14882_ SRi Lankan rice.pdf [file pone.0273386.s002.pdf]
