# Supplementary material for: Sri Lankan traditional parboiled rice: A panacea for hyperglycaemia?
Source: PLoS One. 2022 Sep 15;17(9):e0273386. doi: 10.1371/journal.pone.0273386 (PMC9477285; doi:10.1371/journal.pone.0273386)
Supplement: Attachment — Submitted filename: Response to Reviewers.docx [file pone.0273386.s003.docx]

**Response to Journal and reviewer comments**

|  | Comment | Author response |
| --- | --- | --- |
|  | **Comments of Reviewer #1** | Thank you for the comments which helped to improve and correct contents of this manuscript. |
| 1 | Spelling and insertion in lines 38 & 45 | 38 –corrected as “World’s”  46 - inserted the term “(NCDs)” |
| 2 | Line 95 - 350-400 mL of water for how much rice ? | Included in (350-500 mL/100 g of rice) (line 93-94) |
| 3 | Line 122 - Include how long after cooking rice was served | Included - 1-1.5 hrs after cooking rice (line 123) |
| 4 | Line 126 - Details of how much blood was collected and how blood was stored before analysis | Included - 100 uL of blood and samples were collected in to tubes containing NaF and analysed within 2hrs of collection. Samples were centrifuged immediately after collecting and serum was separated and kept at 25oC prior to analyses. (lines 128-131) |
| 5 | Line 129 - Include the method of calculating IAUC and reference | The reference included (Reference 21 - FAO/WHO 1998). (line 133)  IAUC was calculated using the **trapezoidal rule i**gnoring the area below the fasting glucose level. |
| 6 | Line 148 - Cooking procedure with respect to time, temperature and water content.  Texture of the cooked rice | This is included under sample preparation. We added that rice was cooked using the household rice cooker (Lines - 92-93)  By tasting the cooked rice and cooking until the rice grain was soft to crushing with thumb and forefinger. No other specific test was used. (Line 94)  The sentence reads as follows after additions.    “Rice samples (dehulled only, polished (4%), parboiled) were washed and cooked (household rice cooker) for 30 to 60 minutes by adding a known amount of water (350-500 mL/100 g rice) as required to reach the cooked texture and taste for each differently processed rice variety.” |
| 7 | Line 151 - | Comment is not appearing in the box. |
| 8 | Line 161 - include “digestible” | Included the term “digestible” |
| 9 | Line 162 – Include photos of all portion sizes of all differently processed varieties (18 nos) | Included the photos of all differently processed rice varieties.  Figure 2 - Portion sizes of differently processed rice  (Figure numbers changed than in previous version)  Earlier as the bar chart clearly illustrated (figures 1a and 1b) the moisture content we included only the portion sizes of 2 varieties. |
| 10 | Line 169 - Table 1 – insert (mean± SD) – follow for all tables where applicable | Inserted “(mean± SD)” in all table headings where relevant – Table 2 & Table 4 |
| 11 | Line 185 -(P≥ 0.05) | (P≥ 0.05) was used to indicate that even though low it is not significant. (line 185) |
| 12 | Line 246 – increased | Corrected as “increased” (line 247) |
| 13 | Line 247 –delete digestion and include absorption | Corrected as indicated (deleted digestion and included absorption) (line 248) |
| 14 | Line 258 - regarding if the volunteers did not consume the portion given ………… | The whole portion given to consume was consumed by all volunteers.  When we obtained their feedback on if the portion size containing 50g digestible carbohydrate was adequate or not adequate or too large to be consumed – the volunteers said that the parboiled portion was much more than they would normally consume. However, for the study they consumed the total portion of rice given. We have reworded the sentence to make it clearer.  “The higher moisture content in cooked parboiled rice (Figure 1) made the portion sizes larger (volume) and according to volunteers this portion was much larger than a normal edible portion. “ (lines 258-259) |
| 15 | Line 274 - n=10 | We had a pool of volunteers to study the 18 samples of rice and standard glucose (thrice) and 10 of them were selected randomly to test each variety of rice. So we give the average of 10 individuals for the GI value. |
| 16 | Lines 288 ; 291 & 294 (Symbol for lager/equal than and smaller/equal than | All 3 Corrected |
| 17 | Line 302 – given | Corrected as “given” |
| 18 | Line 319 - change to low to medium | Corrected as “lower GI compared to raw or polished rice” |
|  | **Comments of Reviewer #2:** | Thank you for the comments which helped to improve and correct contents of this manuscript. |
| 1 | The subject matter of the MS is timely and relevant in view of the rice consumption and diabetes and obesity.  The experimental part is voluminous as the data generated is on 6 cultivars and 3 processing variants. The GI is determined on mixed meal instead of single cereal namely the rice. | We highly appreciate the positive comment. |
| 2. | 100% brown rice and just 4% DM rice really does not make a significant difference, because, 4% DM rice also contains some amount of bran. It would have been better if they had polished the rice 8-10% which is the normal DM of the market samples. | Traditional rice is usually available in Sri Lanka as 100% brown rice. Since many nutrients are found in the bran we used 4% polished level.  In future studies will take this into consideration and will include 8-10% polished level as well. |
|  | 3. Parboiling method is not a standard method, as usually, paddy is soaked in cold or hot water till it attains 25 - 35%, then subjected to live steam till the starch completely gelatinises and then dried.  In the present study, the paddy is soaked in boiling water and heated till the grains were split and dried. The method may have also influenced the GI value.  The parboiled paddy was just dehusked (brown rice). It will have poor cooking quality as the bran hinders swelling of the grains during cooking. The authors should clarify these aspects. | We used not the industrial method that is generally utilized but the household method that is used in Sri Lanka to make parboiled rice at village levels. The consumption of parboiled rice is very less in Sri Lanka and one intention of this study was to demonstrate that parboiled rice is very much suitable to prevent or control the high prevalence of NCDs in our country.  Like mentioned earlier traditional rice is consumed as brown rice (100%). However, when freshly cooked rice was taken parboiled rice had the highest level of moisture compared to polished and unpolished raw rice (pls check Figure 1).  However, the cooking time and the water required to cook parboiled rice varieties were 50 to 60 minutes and 480 to 500 mL of water respectively and more than required for raw or raw polished. |
| 4. | The language of the MS is far from satisfactory wrt the use of appropriate scientific terminologies (eg L No. 23, Sri Lankan traditional rice produce lower glycaemic responses, instead it should be Sri Lankan traditional rice elicit lower glycaemic responses, L. No. 147, varieties are stated in tables, it should be varieties are presented in tables ). | Corrections have been adhered (line 23, 152, 251) (for all tables) and  manuscript has been corrected for grammar. These corrections were not highlighted as there were many corrections. (Thank you) |
| 5 | Digestible starch normally reduces on parboiling but in the case of Dharnala sample it has increased ? | Yes. However, the increase in parboiled rice is not significant to that of raw rice.  This is an observation we checked repeating the assay many times. Probably this could be due to the higher protein content of *Dahanala* (Table 1) which can affect the starch digestibility. |
| 6 | The total DF values are considerably on higher side? | Yes, for Raw and Parboiled rice of these varieties. Since raw polished value is lower we presume that this is due to bran and RS which increased following cooking |
| 7 | The portion size of rice used for GI for parboiled rice is 1.5 to 2 times higher compared to raw as well as milled rice. How it is possible. This need satisfactory explanation. | Parboiled rice contained high amount of moisture compared to other 2 varieties (Figure 1). Thus the volume of the portion of parboiled rice that contained 50 g of digestible carbohydrate was comparatively large. This has been explained in the discussion before Table 3. (lines 256-260) |
|  | **Other corrections to language** |  |
|  | Line 105 – for ash content determination | for determining the ash content |
|  | Line 255 - varied in a wide range as 140g to 412g | Varied widely (140 -412 g) |
|  | Line - Red pericarp rice varieties contain higher content of minerals | Red pericarp rice varieties contain high content of minerals (line 189) |
|  | Throughout the manuscript grammatical errors were corrected. | All were not highlighted. |
|  |  |  |
